# Medicare Advantage Plan Spending and Payments Under the Hospice Carve-Out

**DOI:** 10.1001/jamanetworkopen.2025.27724

**Published:** 2025-08-19

**Authors:** Meghan Bellerose, Andrew M. Ryan, Claire K. Ankuda, David J. Meyers

**Affiliations:** 1 Department of Health Services, Policy, and Practice, Brown University School of Public Health, Providence, Rhode Island; 2 Department of Geriatrics and Palliative Medicine, Icahn School of Medicine at Mount Sinai, New York, New York

## Abstract

**Importance:**

In 2021, the Centers for Medicare & Medicaid Services implemented a Value-Based Insurance Design (VBID) model to test the impact of including hospice services in the Medicare Advantage (MA) benefits package. In December 2024, the VBID was ended following widespread dissatisfaction, signaling a return to the hospice carve-out model. Under the carve-out model, after an MA enrollee elects hospice, health care related to their terminal illness is paid for by fee-for-service (FFS) Medicare. MA plans stop receiving the inpatient and outpatient portions of that enrollee’s capitated payment but continue to receive premium and rebate payments.

**Objective:**

To estimate MA plan spending relative to premium and rebate payments for hospice enrollees under the carve-out model.

**Design, Setting, and Participants:**

This cross-sectional study estimated MA plan spending, premiums, and rebate payments for MA enrollees who elected hospice from 2017 through 2019 using 2016 to 2020 FFS claims, MA encounter records, and publicly available plan-level data.

**Main Outcomes and Measures:**

MA plan spending and net payments for MA enrollees who elected hospice. Excess payments to MA plans following hospice election were estimated as the difference between MA plan spending and payments. Analyses were conducted between May 2024 and June 2025.

**Results:**

We included 314 087 MA enrollees (180 914 females [57.6%]; mean [SD] age, 80.7 [10.0] years); 125 321 (39.9%) were aged 85 years or older. The mean (SD) length of hospice stay was 1.76 (2.49) months. In the year after hospice election, mean MA spending fell to $57 per enrollee per month, with 81% of enrollees having no inpatient, outpatient, physician, skilled nursing facility, home health care, or prescription drug expenses that MA plans were liable for. MA plans continued to receive $120 per enrollee per month in premiums and rebates, equating to $23 million to $58 million in excess payments to MA plans annually under scenarios in which either 50% of rebate payments or 0% of rebate payments were allocated toward supplemental benefits, respectively.

**Conclusions and Relevance:**

In this cross-sectional study, MA plans received high premium and rebate payments for beneficiaries enrolled in hospice despite low health care spending after enrollees elected hospice. To reduce excess payments, the Centers for Medicare & Medicaid Services could require MA plans to submit information on enrollees’ use of supplemental benefits and adjust payments made after election of hospice to align with spending.

## Key Points

**Question:**

Do Medicare Advantage (MA) plans receive excess payments for beneficiaries enrolled in hospice under the hospice carve-out model?

**Findings:**

In this cross-sectional study, with 314 087 MA enrollees, MA plan payments exceeded plan spending for most beneficiaries following hospice election from 2017 to 2019, equating to an estimated $23 million to $58 million in excess payments to MA plans per year.

**Meaning:**

In this study, MA plans continued to receive high premiums and rebate payments for beneficiaries enrolled in hospice despite low MA health care spending following hospice election.

## Teaser

This cross-sectional study estimated the excess payments to Medicare Advantage for beneficiaries enrolled in hospice under the hospice carve-out model.

## Introduction

Use of the Medicare hospice benefit has increased over time, from 22.9% of Medicare decedents in 2000 to 51.6% in 2023.^1,2^ Hospice coverage includes palliative and supportive care focused on improving the quality of life of individuals who have been given a prognosis of less than 6 months to live.^1^ After a beneficiary elects hospice, Medicare no longer covers curative treatment for their terminal illness but will continue to cover services for conditions unrelated to the terminal illness.

Hospice is a carved-out benefit of the Medicare Advantage (MA) program, meaning that it is not included in the MA benefits package. Under the carve-out model, an MA enrollee who elects hospice remains in their MA plan, but their hospice services are paid by fee-for-service (FFS) Medicare.^3^ Part A (inpatient) and Part B (outpatient) services and Part D prescription medications unrelated to their terminal illness continue to be paid by the MA plan. As a result of this structure, FFS Medicare spends more on hospice enrollees’ care compared with what MA plans spend, necessitating adjustments to how MA plans are paid for enrollees who elect hospice.

MA plans are paid for each enrollee based on the relationship between their annual bid, an estimate of their costs to cover Part A and B services for an average enrollee, and a benchmark.^4^ Benchmark payments in MA are derived from county-level FFS spending for Parts A and B, with adjustments for geographic variation, growth, and plan quality. FFS hospice spending is excluded from this calculation; thus, the benchmark captures high end-of-life spending, including spending for beneficiaries who die without electing hospice. Spending is averaged across the entire risk pool, meaning that MA plans are compensated through benchmark payments that account for their likelihood of covering high-cost enrollees.

To account for MA plans’ lowered contributions toward enrollees’ health care during hospice, MA plans stop receiving the Part A or Part B portions of enrollees’ capitated payments upon hospice election. However, MA plans continue to receive monthly premiums paid by enrollees to account for health care spending unrelated to enrollees’ terminal illnesses and rebate payments, which MA plans may use to reduce out-of-pocket costs or provide supplemental benefits, such as vision, hearing, and dental care.^4^

In 2021, the Centers for Medicare & Medicaid Services (CMS) implemented an MA Value-Based Insurance Design (VBID) model to test the impact of including the Medicare hospice benefit in the MA benefits package. The goal of this change was to promote integrated, coordinated care by giving MA plans responsibility for the full continuum of care. In March 2024, after receiving feedback about operational challenges, administrative burden, declining participation among MA organizations, and unclear quality improvements or cost-savings for enrollees,^5,6^ CMS announced plans to end the MA hospice benefit in December 2024 and return to a carve-out model.^7^

With the hospice carve-out reinstated, it is important to understand whether the existing payment structure facilitates appropriate payment to MA plans for hospice enrollees’ care. Continued premium and rebate payments represent a source of potential excess payment to MA plans if they are not aligned with the use of health services or use of supplemental benefits in the last days or months of life. We estimated MA plan spending on enrollees who began hospice from 2017 through 2019 and then calculated excess payments to MA plans for hospice enrollees as the difference between MA plan spending and payments.

## Methods

### Data and Study Sample

This analysis used 2016 to 2020 Medicare Master Beneficiary Summary Files, MA encounter records, and FFS Medicare claims from a 20% random national sample of FFS and MA enrollees. Our study sample included beneficiaries who elected hospice from January 1, 2017, through December 31, 2019, while enrolled in an MA plan and living in a US state or the District of Columbia (eFigure in Supplement 1). To provide a comparison to FFS spending on FFS Medicare enrollees, we also evaluated an equivalent sample of beneficiaries who elected hospice while enrolled in FFS Medicare. This study was deemed exempt from review and the requirement for informed consent by Brown University’s Institutional Review Board and adheres to the Strengthening the Reporting of Observational Studies in Epidemiology (STROBE) reporting guideline for observational studies.

### Estimating FFS and MA Spending

We estimated spending for each beneficiary from the day after hospice election through 12 months after hospice election or until death, discharge from hospice, or disenrollment from Medicare. MA records do not contain reliable spending information.^8^ Therefore, following previous work, we used the 2016 to 2020 FFS fee schedules to approximate prices in MA.^9^ This approach operates under the assumption that spending by MA plans is roughly equivalent to what would be spent by FFS on the services received by the beneficiary were they not enrolled in MA; in other words, applying FFS prices to MA utilization patterns.^10^

To summarize this approach, we first estimated MA spending on inpatient, outpatient, and physician services by calculating mean FFS spending per diagnosis-related group (DRG) or Healthcare Common Procedure Coding System (HCPCS) code and year. We excluded claims from people who were not enrolled in FFS Medicare for the entire year and claims with a negative payment value. We then merged mean FFS spending values by DRG and HCPCS code to corresponding DRG codes and HCPCS codes within 2016 to 2020 inpatient, outpatient, and physician MA encounter records from our study population.

Next, to estimate MA spending on skilled nursing facility and home health care, we created models estimating FFS spending based on enrollee characteristics from the Minimum Data Set (MDS) and the Outcome and Assessment Information Set (OASIS), as reimbursement varies based on patient characteristics and health status. MDS and OASIS are health assessments required for residents of Medicare- and Medicaid-certified nursing facilities and those receiving home health care. During our study period, assessments were available for 49% to 75% of residents, with more assessments completed each year.^11^ We applied the models to MA encounter records from our study population linked to MDS and OASIS responses to assign each claim an estimated price based on the enrollee’s characteristics and health status. When a beneficiary in MA elects into hospice, they generate hospice claims in the FFS hospice files, which include spending values. Part D spending values are recorded directly in enrollment files for MA enrollees. We calculated FFS spending for our comparison sample by summing FFS payments reported on inpatient, outpatient, physician, skilled nursing facility, home health care, Part D prescription drug, and hospice claims.

### Estimating Premium and Rebate Payments

We estimated monthly premium payments for each MA enrollee using data from the CMS Descriptions of the Drug Plans files, which contain mean premiums by MA plan identification number, county, and year.^12^ We estimated monthly rebate payments for each MA enrollee using CMS Part C Plan Payment Data, which contain rebates by MA plan identification number and year.^13^

### Estimating Excess Payments to MA Plans

We estimated excess payments to MA plans per enrollee as the difference between MA plan spending and premium plus rebate payments. We produced annual estimates by multiplying these values by the mean length of hospice stay and by 5 to approximate a 100% national sample from our 20% national sample. We calculated the length of hospice stay as the total number of days that an individual was enrolled in hospice and MA, including nonconsecutive stays (eg, discharge from hospice and subsequent re-enrollment).

MA encounter data lack information on supplemental benefit use. To address this data gap, we present results under 2 scenarios: if 50% of rebate payments were used to provide supplemental benefits to enrollees following hospice election and if no rebate payments were used. We selected 50% as our upper feasible limit, as 50% of MA enrollees in our sample died within the first 30 days of hospice election. The Medicare hospice benefit provides direct coverage for over-the-counter medications related to one’s terminal illness and transportation services, precluding the need for MA to use rebate dollars to provide those benefits to hospice enrollees, and the most commonly offered supplemental benefits, including vision, hearing, fitness, and dental benefits are unlikely to be used by enrollees in the last month of life.^3^

### Statistical Analysis

We used SAS version 9.4 to identify our sample and Stata version 18.0 for the analysis. We conducted the analysis from May 2024 through June 2025.

## Results

### Sample Characteristics

**Figure 1.**
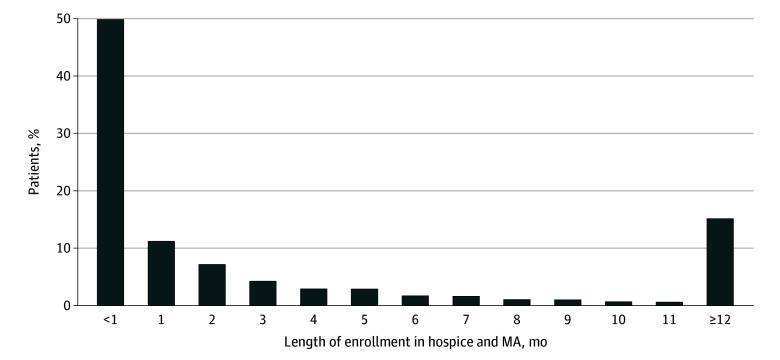
Length of Hospice Stays Among Medicare Advantage Beneficiaries Who Entered Hospice From 2017 to 2019

Our MA sample included 314 087 beneficiaries (180 914 females [57.6%] and 133 173 males [42.4%], mean [SD] age, 80.74 [10.04] years) who elected hospice while enrolled in an MA plan, including 94 984 who elected hospice in 2017, 104 813 in 2018, and 114 290 in 2019. Across all years, 125 321 enrollees (39.9%) were aged 85 years or older, 105 533 (33.6%) were aged 75 to 84 years, and 19 159 (6.1%) were younger than 65 years (Table 1). A total of 73 496 beneficiaries (23.4%) were dual enrolled in Medicaid; 15 704 (5.0%) switched from MA to FFS Medicare. The mean (SD) length of hospice among those who remained enrolled in MA was 54 (76) days (1.8 months); 157 672 (50.2%) remained enrolled in hospice for at least 1 month, 99 880 (31.8%) for at least 3 months, 68 471 (21.8%) for at least 6 months, and 48 055 (15.3%) for at least 12 months (Figure 1). Our comparison FFS sample included 579 917 beneficiaries who elected hospice while enrolled in FFS Medicare and had characteristics similar to those in the MA sample with the exception that a larger percentage were younger than age 65 (46 973 [8.1%]) (eTable in Supplement 1). The mean (SD) length of hospice among those who remained enrolled in FFS was 52 (75) days (1.7 months) (eFigure 2 in Supplement 1).

**Table 1.** Characteristics of Medicare Advantage Enrollees Entering Hospice From 2017 to 2019

Characteristic	Hospice enrollees by year, No. (%)			
	2017 (n = 94 984)	2018 (n = 104 813)	2019 (n = 114 290)	2017-2019 (N = 314 087)
Age category, y				
<65	5699 (6.0)	6289 (6.0)	7086 (6.2)	19 159 (6.1)
65-74	19 377 (20.4)	21 591 (20.6)	23 315 (20.4)	64 074 (20.4)
75-84	32 010 (33.7)	34 903 (33.3)	38 744 (33.9)	105 533 (33.6)
≥85	37 899 (39.9)	42 030 (40.1)	45 259 (39.6)	125 321 (39.9)
Sex				
Female	54 711 (57.6)	60 372 (57.6)	65 831 (57.6)	180 914 (57.6)
Male	40 273 (42.4)	44 441 (42.4)	48 459 (42.4)	133 173 (42.4)
Dual-enrolled in Medicare and Medicaid	21 561 (22.7)	24 526 (23.4)	27 430 (24.0)	73 496 (23.4)

### Spending, Premiums, and Rebate Payments

**Figure 2.**
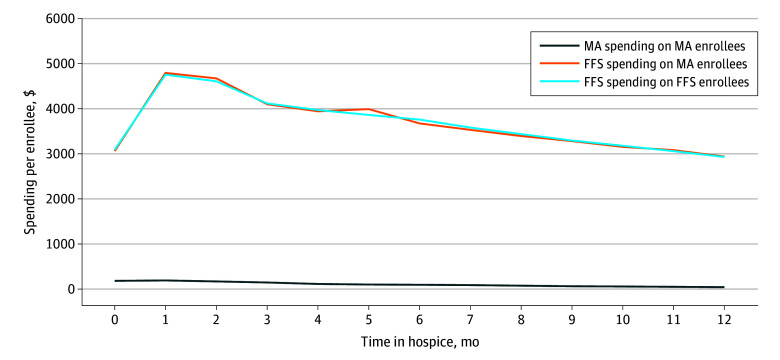
Mean Estimated Spending on Health Services by MA Plans and FFS Medicare for Beneficiaries Entering Hospice From 2017 to 2019 by Months Since Hospice Election MA indicates Medicare Advantage; FFS, fee for service.

Across the 12 months following hospice election, 80.5% of MA enrollees had no spending on care unrelated to their terminal illness that MA plans were liable for. Estimated MA spending on hospice enrollees during this period was $57.09 per beneficiary per month, including a mean (SD) of $34.43 ($42.78) spent on inpatient, outpatient, skilled nursing home, and home health care claims and $22.66 ($27.43) spent on Part D claims. Mean (SD) FFS spending on MA enrollees’ hospice services during this period was $2108.17 ($1697.88) per beneficiary per month. In comparison, mean (SD) FFS spending on FFS enrollees’ hospice services and care unrelated to their terminal illnesses was $2099.37 ($1693.37) per beneficiary per month across the 12 months following hospice (Figure 2). From 2017 through 2019, MA plans were paid a mean (SD) of $43.86 ($53.55) in premiums and $76.25 ($71.25) in rebates per beneficiary per month ($120.11 total) for beneficiaries actively enrolled in hospice (Table 2).

**Table 2.** Estimated MA Plan Payments and Spending for the First 12 Months After Beneficiaries Entered Hospice

Year	MA beneficiaries entering hospice		Plan payment^a^		Plan spending^b^		Net payments^c^	
	Estimated No.	Hospice duration, mean (SD), mo	Premiums per beneficiary per month, mean (SD), $	Rebates per beneficiary per month, mean (SD), $	Parts A and B spending on care unrelated to hospice per beneficiary per month, mean (SD), $	Part D spending per beneficiary per month, mean (SD), $	If 50% of rebates are spent on supplemental benefits, $	If 0% of rebates are spent on supplemental benefits, $
2017	474 920	1.73 (2.47)	49.21 (57.02)	71.00 (69.16)	40.95 (41.61)	29.32 (29.93)	11 864 072	41 031 283
2018	525 065	1.76 (2.48)	45.70 (55.56)	75.67 (70.50)	31.45 (42.50)	24.64 (30.02)	25 362 320	60 326 188
2019	571 450	1.79 (2.51)	37.72 (47.80)	81.47 (73.27)	30.88 (45.14)	14.02 (19.87)	34 323 259	75 990 907
2017-2019	1 570 435	1.76 (2.49)	43.86 (53.55)	76.25 (71.25)	34.43 (42.78)	22.66 (27.43)	68 808 924	174 185 112

Abbreviation: MA, Medicare Advantage.

^a^ Premiums were estimated using the Centers for Medicare & Medicaid Services Descriptions of the Drug Plans Files. Rebates were estimated using Centers for Medicare & Medicaid Services Part C Plan Payment Data.

^b^ Estimates include spending on inpatient, outpatient, physician, hospice, skilled nursing facility, home health care, and Part D claims.

^c^ Net payments reflect MA plan payments minus MA plan spending multiplied by the number of hospice enrollees and the duration of hospice enrollment.

### Excess Payments to MA Plans

In the year after hospice election, estimated MA spending was $57.09 per beneficiary per month, and MA plans were paid an estimated $120.11 per beneficiary per month for a net of $63.02 per beneficiary per month. If 50% of rebate payments were used to provide supplemental benefits to hospice enrollees, this would result in an excess payment of $68 808 924 over a 3-year period or $22 936 308 per year. If no rebates were used to provide supplemental benefits to hospice enrollees (0% scenario), this would result in an excess payment of $174 185 112 over a 3-year period or $58 061 704 per year. Excess payments were highest in 2019 at $34 323 259 under the 50% scenario and $75 990 907 under the 0% scenario.

## Discussion

In this national, cross-sectional study, we compared MA plan spending with plan payments for beneficiaries enrolled in hospice under the Medicare hospice carve-out model. We found that following hospice election, mean MA plan spending declined to $57 per enrollee per month, and 80% of MA enrollees did not receive any health services that MA plans were liable for. During this period, plans continued to receive premium and rebate payments that exceeded spending, equating to an estimated $23 to $58 million in excess payments to MA plans per year under scenarios in which 0% to 50% of rebate dollars were used to provide supplemental benefits to enrollees at end-of-life. These payments are not tied to service delivery or the medical risk the plan bears during the hospice period, and their continued flow raises questions about alignment between MA plan payments and value.

For several reasons, our analysis may underestimate contemporary excess payments to MA plans resulting from the hospice carve-out model. First, because we only tracked hospice and MA enrollment for 12 months following hospice election, our length of hospice stay values were lower than national averages. Between 2017 and 2019, the mean length of stay in our sample was 54 days, while the national mean was 91 days.^14^ Although the median length of stay was stable across this period at 18 days (both within our sample and nationally), mean length of stay increased each year due to growth in the number of stays longer than 6 months.^2,14^ Second, since 2019, mean MA rebate payments have increased 81% from $107 to $194 per enrollee per month.^1^ While Medicare spending also increased by roughly 41% over this period, and national MA premiums fell by 43%, the increase in rebate dollars has resulted in higher net payments to MA plans.^15,16^ If we apply these length of stay, rebate, premium, and spending adjustments to our 2019 estimates, overpayment to MA plans increases from $34 to $46 million per year under the 50% scenario and $76 million to nearly $174 million per year under the 0% scenario.

While it is possible that at least 50% of rebate payments are allocated toward supplemental benefits for hospice enrollees, the estimate generated under the 0% scenario may be closer to the true value. It is unlikely that rebate payments are used to provide supplemental benefits to MA beneficiaries to the same degree before and after hospice election, and among the full MA population, evidence suggests that supplemental benefits use is low and that enrollees lack information on which supplemental benefits they have access to and how to use them.^17,18,19^ From 2017 through 2021, MA enrollees did not report more supplemental benefits use compared with traditional Medicare enrollees and paid 9.0% and 9.3% less out of pocket for eyeglasses and dental visits, respectively, but no less for other supplemental services.^17^

Given the rapid growth of MA enrollment (17%-53% in 2000-2024), broad dissatisfaction with the hospice VBID, and the degree of excess payments to MA plans under the current system, it is worth considering reforms to hospice payment in MA.^1^ Rather than eliminate rebate payments, which could lower the incentive for plans to offer nonmedical benefits to hospice enrollees, CMS could require plans to report on the extent to which supplemental benefits were used by this population and perform a corresponding actuarial adjustment to reimburse plans appropriately for services provided. This type of adjustment could become more feasible in coming years following CMS’ announcement that they will require plans to report enrollees’ use of supplemental benefits in encounter records beginning in 2024.^20,21^ This policy could also incentivize plans to facilitate use of supplemental benefits among hospice enrollees. Benefits such as meal services, bathroom safety devices, in-home supports, and support for caregivers (included in 65%, 24%, 6%, and 5% of MA plans in 2025, respectively) could be beneficial to hospice enrollees and their families.^22^

Second, CMS could require MA plans to lower Part D premiums following hospice election to account for declines in spending. Our results indicate that following hospice election, MA beneficiaries continue to use Part D prescription drugs unrelated to their terminal illnesses that MA plans are liable for, but at a far lower level than their pre-hospice use ($283 per beneficiary per month in the 12 months before hospice compared to $23 per beneficiary per month in the 12 months after).

As an important caveat to these recommendations, under the carve-out model, lowering MA plan payments for hospice enrollees could create perverse incentives for MA plans to discourage high-cost end-of-life patients from electing hospice so that they continue to drive up plan risk scores and payments. Should changes be made to the MA hospice payment system, it would be important to monitor corresponding trends in hospice election.

### Limitations

This study has limitations. While most prior research has found that MA prices are very similar to FFS prices,^10^ we cannot determine MA spending perfectly using current data and may be missing data on subcapitated payments that plans make to providers, which are not currently captured in encounter records. In addition, our MA spending estimates do not account for geographic variation in spending. We did not include pre-hospice spending on durable medical equipment, as we did not have access to those claims; however, following hospice election, durable medical equipment is covered by FFS Medicare under the hospice benefit. Also, as noted above, MA plans do not currently report on the extent to which they use rebate payments to provide supplemental benefits to hospice enrollees or lower premiums. We addressed this limitation by estimating net payments to MA plans under 2 rebate use scenarios.

## Conclusions

Under the hospice carve-out model, MA plans continue to receive high premium and rebate payments for beneficiaries enrolled in hospice despite steep declines in those beneficiaries’ MA health care spending following hospice election. To reduce overpayments to MA plans resulting from the hospice carve-out, CMS could require MA plans to report hospice enrollees’ use of supplemental benefits and use that information to align monthly MA plan payments with expenditures.

## Notes

Supplement 1. **eFigure.** Flow diagram of study inclusion **eTable.** Characteristics of fee-for-service Medicare enrollees entering hospice between 2017-2019

Supplement 2.
Data Sharing Statement
