# Supplementary material for: Medicare Advantage Plan Spending and Payments Under the Hospice Carve-Out
Source: JAMA Netw Open. 2025 Aug 19;8(8):e2527724. doi: 10.1001/jamanetworkopen.2025.27724 (PMC12365699; doi:10.1001/jamanetworkopen.2025.27724)
Supplement: Supplement 1. — eFigure. Flow diagram of study inclusion eTable. Characteristics of fee-for-service Medicare enrollees entering hospice between 2017-2019 [file jamanetwopen-e2527724-s001.pdf]

Supplemental Online Content

Bellerose M, Ryan AM, Ankuda CK, Meyers DJ. Medicare Advantage Plan Spending and Payments Under the Hospice Carve-Out. *JAMA Netw Open.* 2025; 8(8): e2527724. doi: 10.1001/jamanetworkopen.2025.27724

eFigure. Flow diagram of study inclusion
eTable. Characteristics of fee-for-service Medicare enrollees entering hospice between 2017-2019

This supplemental material has been provided by the authors to give readers additional information about their work.

© 2025 Bellerose M. et al. JAMA Network Open.

eFigure 1. Flow diagram of study inclusion

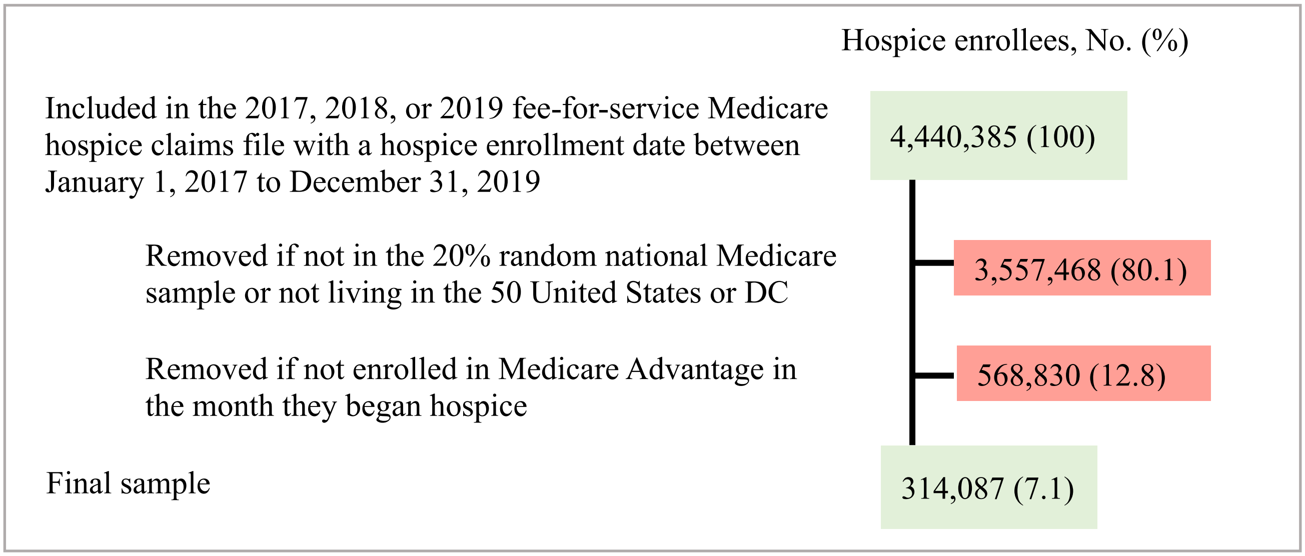

*We did not remove individuals with missing data from the analysis.

© 2025 Bellerose M. et al. JAMA Network Open.

eTable 1. Characteristics of fee-for-service Medicare enrollees entering hospice between 2017-2019

	Hospice enrollees, No. (%)			
Year	2017	2018	2019	2017-2019
Total N	192,159	193,039	194,719	579,917
Characteristic				
Age category, years				
<65	15,757 (8.2)	15,636 (8.1)	15,578 (8.0)	46,973 (8.1)
65-74	36,318 (18.9)	37,643 (19.5)	37,775 (19.4)	111,924 (19.3)
75-84	58,416 (30.4)	58,491 (30.3)	59,389 (30.5)	176,295 (30.4)
85 plus	81,668 (42.5)	81,269 (42.1)	81,977 (42.1)	244,725 (42.2)
Female	112,259 (58.4)	112,117 (58.1)	113,229 (58.2)	337,628 (58.2)
Dual enrolled in Medicaid	45,964 (23.9)	45,905 (23.8)	46,713 (24.0)	138,600 (23.9)

© 2025 Bellerose M. et al. JAMA Network Open.
