# Supplementary material for: Medicare Advantage Plan Spending and Payments Under the Hospice Carve-Out
Source: JAMA Netw Open. 2025 Aug 19;8(8):e2527724. doi: 10.1001/jamanetworkopen.2025.27724 (PMC12365699; doi:10.1001/jamanetworkopen.2025.27724)
Supplement: Supplement 2. — Data Sharing Statement [file jamanetwopen-e2527724-s002.pdf]

Data Sharing Statement

Bellerose. Medicare Advantage Plan Spending and Payments Under the Hospice Carve-Out. *JAMA Netw Open*. Published August 19, 2025. doi:10.1001/jamanetworkopen.2025.27724

Data
Data available: No

Additional Information
Explanation for why data not available: The claims data used in this analysis were provided by CMS under a data use agreement
